# Dietary Acculturation among Filipino Americans

**DOI:** 10.3390/ijerph13010016

**Published:** 2015-12-22

**Authors:** Persephone Vargas, Leo-Felix Jurado

**Affiliations:** Department of Nursing, William Paterson University 300 Pompton Rd., Wayne, NJ 07470, USA; juradol@wpunj.edu

**Keywords:** acculturation, dietary acculturation, Filipino diet, Filipino nutrition, Filipino immigrants, Filipino Americans

## Abstract

Acculturation, the subsequent changes that occur in one culture after continuous first hand contact with another culture, impacts the dietary habits and health risks of individuals. This study examines the acculturation, dietary habits and anthropometric measurements in a sample of 210 first generation Filipino American immigrants in New Jersey (NJ). Acculturation was measured using the Short Acculturation Scale for Filipino Americans (ASASFA). Dietary acculturation was measured using the Dietary Acculturation Questionnaire for Filipino Americans (DAQFA) and dietary intake was determined using the Block’s Brief Food Frequency Questionnaire (BFFQ). Anthropometric measurements were obtained including weight, height and waist circumference. Acculturation had a significant negative relationship with Filipino Dietary acculturation. Western dietary acculturation was significantly correlated with caloric intake (r(208) = 0.193, *p* < 0.01), percentage fat intake (r(208) = 0.154, *p* < 0.05), percentage carbohydrate intake (r(208) = −0.172, *p* < 0.05), Body Mass Index (BMI) (r(208) = 0.216, *p* < 0.01) and waist circumference (r(208) = 0.161, *p* < 0.01). There was no significant correlation between Filipino dietary acculturation, dietary intake and anthropometric measurements. The results showed that Filipino American immigrants have increased risks including increased BMI, waist circumference and increased fat intake. Over all, this research highlighted some dietary changes and their effects on dietary intake and health status.

## 1. Introduction

Acculturation is a complex concept that affects health behaviors among all immigrants. Acculturation occurs when an individual migrates to a new country and goes through a process of adapting to the host country. The process can result in several challenges and changes that can strongly impact the immigrant’s health positively or adversely [1]. A significant aspect of the acculturation process is the adaptation of the dietary practices of the host country, also called dietary acculturation [2].

In dietary acculturation, immigrants may find new ways to use traditional food, exclude other food and/or consume “new” food. The changes in the immigrant’s dietary pattern in the United States (U.S.) include: increased consumption of energy-dense and processed food, decrease in fruits, vegetables and whole grains, increase in portion sizes and replacement of drinks to sweetened beverages. This adoption of less healthy American food can lead to an increase in obesity and other health related problems [2].

There is an estimated 3.7 million Filipinos in the U.S., making them the third largest Asian subgroup [3]. Filipinos are also the fourth largest immigrant group, with 1.8 million born outside of the U.S. [3]. Like all other immigrants, Filipino Americans experience acculturative changes. There are only a few studies that have examined the effects of acculturation on health behaviors among Filipino American immigrants. The results of the current literature review of acculturation provide support on the impact of acculturation on Filipino immigrants’ health outcomes. The increased prevalence of hypertension (HTN) among Filipino Americans has been well documented [4,5,6]. Migration and length of residency in the U.S. have been found to have significant correlation with hypertension among Filipino immigrants [7,8]. Recent studies have documented that Filipino Americans have a HTN prevalence rate of 51%–53%, the highest HTN prevalence among all racial/ethnic groups [8,9]. Studies have also shown that Filipino American men have the second highest prevalence rate of diabetes (15.8%) among all ethnic races in the U.S. [10].

### 1.1. Acculturation and Anthropometric Measurements

Increased BMI and abdominal obesity increase the risks for chronic diseases such as cardiovascular disease and diabetes. The effect of acculturation on BMI has been documented among Filipino Americans. Asians have a lower BMI than Caucasians [11], however, studies have also shown that Filipinos have a higher BMI among other Asian American subgroups [12,13] and U.S.-born Filipino-Americans have a higher BMI compared to Filipino immigrants [14].

Overweight and obesity were significantly higher in Filipino immigrant women living in the U.S. compared to the Filipino women in the Philippines. The percentage of Filipino women with a BMI of ≥25 in the Philippines was 28.8% as compared to Filipino immigrant women in San Diego which was 49.2% and in Hawaii 50.5% [7]. In addition, Filipino women have a significantly higher visceral adiposity compared to Caucasians and African Americans, even in women with normal weight and waist circumference [15].

### 1.2. Dietary Changes among Filipino American Immigrants

Studies on dietary patterns among Filipino immigrants show changes that may explain the increase in obesity and diet-related chronic disease rates among the Filipino immigrants. Dietary changes among Filipino immigrants include the addition of milk, increased consumption of meat, fruits and fresh vegetables (salads), less starchy foods and snacks. An earlier study identified an increase in caloric intake, with twice as many calories from protein and three times as many from fat [16]. Other dietary changes noted in other studies include increase in meat, dairy, bread, fat and sugar intake [17,18].

Serafica *et al.* [18] explored the relationship between dietary acculturation, fat and sugar intake and anthropometric measurements. The results showed that the Filipino Dietary Acculturation scale was a significant negative predictor of acculturation. The participants with a higher consumption of traditional food had a lower acculturation score. However, the Western Dietary Acculturation scale was not a significant predictor of acculturation. In addition, participants who had a higher intake of fat and sugar were significantly correlated with having lower Filipino Dietary Acculturation or having lower traditional Filipino food intake. Conversely, a higher fat and sugar intake was a strong predictor of Western Dietary Acculturation or having a higher Western food intake. Increased fruit and vegetable intake was also found to be significantly related to Filipino Dietary Acculturation but not to Western Dietary Acculturation. Acculturation and Filipino Dietary Acculturation were not found to be predictors of anthropometric measurements. The study showed that the most important positive predictors of increased BMI and waist-hip ratio were the Western Dietary Acculturation Scale and sugar and fat intakes.

### 1.3. Study Purpose

Despite the alarming increase in the health risks and health status of Filipino Americans, there is sparsity in studies investigating the effects of acculturation on dietary patterns and diet-related health indicators, such as BMI and waist circumference. The present study aims to examine the acculturation level and dietary pattern among first generation Filipino American immigrants and investigate the relationship among these variables with dietary-related health indicators.

The following questions were examined:

(1). What is the level of acculturation and dietary acculturation of the Filipino American immigrants in NJ?
(2). Is there a relationship between level of acculturation and dietary acculturation?
(3). Is there a relationship between acculturation, dietary acculturation and dietary intake?
(4). Is there a relationship between acculturation, dietary acculturation and anthropometric measurements?

Based on the existing literature, we hypothesized that a higher acculturation level will decrease Filipino dietary acculturation and will increase dietary intake (caloric, fat and carbohydrate intake) and anthropometric measurements. We expected Filipino dietary acculturation to have a significant negative relationship with dietary intake and anthropometric measurements. Lastly, we postulated that Western dietary acculturation will have a significant positive relationship with dietary intake and anthropometric measurements.

## 2. Methods

### 2.1. Study Design and Participants

This study was conducted in NJ the fifth state with the largest Filipino immigrants, with an estimated 110,650 Filipino immigrant residents [4]. Approximately half of the Filipino population resides in three counties: Hudson, Bergen (Northern NJ) and Middlesex (Central NJ) [19].

A cross-sectional study design was used and included a convenience sample of 210 first generation Filipino American immigrants. The inclusion criteria for study participation were: self-identified first generation Filipino American immigrants, over the age of 18 and those who could speak and read English. The exclusion criteria were: pregnant women, those with medical conditions that required a prescribed therapeutic diet and those with less than one year of residency in the U.S.

The sample size was determined using a sample size calculator [20]. A power analysis using a confidence level of 95% and confidence interval of seven revealed *N* = 196 [21,22]. Initially, 215 individuals participated in the study. Of these, 210 completed the survey, giving a response rate of 98%.

### 2.2. Procedure

Institutional Review Board (IRB) approval was obtained on 20 May 2014 from William Paterson University. Data collection was conducted from June 2014 to August 2014. The participants were recruited from Filipino organizations, churches and festivals in New Jersey. The leaders of community organizations, including religious, civic, professional, social groups and alumni associations were identified through referrals. Initial contact with the identified community leaders was done through email and telephone conversations to explain the purpose of the study and the recruitment of participants. A follow-up written communication was sent indicating the nature and purpose of the study. Once the leader agreed to participate, one of the authors (PV) was introduced to the organization members.

Five church-based and two Filipino-American Festivals were identified for data collection. A schedule was determined through consultation with the community leaders and took place after their organization meeting or church service and during the festivals. Snacks were provided during data collection at church meetings. At the Filipino-American festivals, $10 Target gift cards were provided as incentive for participation.

The purpose of the study was verbally explained to the participants, as a group or individually. All participants were asked to sign an informed consent prior to participation. The participants answered the questionnaires which took approximately 30–45 min to complete. To ensure anonymity, identifying personal information were not obtained and the questionnaires were coded.

Once the questionnaires were completed, the participant’s anthropometric measurements were obtained. The measurements included weight, height and waist circumference. During the measurement, privacy was provided by using either a separate room or a privacy curtain. Anthropometric measurements were recorded at the end of the questionnaires by the researcher.

### 2.3. Study Measures

#### 2.3.1. Socio-Demographic Questionnaire

The following socio-demographic were assessed: gender, age, employment status, occupation, income, marital status and years of education. Migration-specific data were obtained, including years of residency in the U.S. and age of migration to the USA

Factors influencing dietary acculturation were also evaluated: the perception of availability and affordability of traditional food. The questions included were: “Are you able to obtain Filipino food or ingredients easily” and “Do you think Filipino food/ingredients are affordable?” Both questions were answerable with “yes” or “no”.

#### 2.3.2. Acculturation

A Short Acculturation Scale for Filipino Americans (ASASFA) was used to determine acculturation level. The ASASFA is a validated twelve-item questionnaire that determines a Filipino American’s level of acculturation. It was derived from the Short Acculturation Scale for Hispanics. The tool measures three factors of acculturation in a 5-point Likert scale: language use, media language preference and ethnic social relation [23].

Each item is scored according to the value assigned to the response. The lowest total score is 12 and the highest total score is 60. The possible mean scores for the total scale and subscales range from 1–5. The higher mean scores indicate a higher level of acculturation toward the American culture and the lower mean scores indicate less acculturation. The scale also permits classification as “bicultural,” indicating that a person has adapted both Filipino and American preferences [24]. The ASASFA has been used in other studies and has an established Cronbach’s alpha coefficient of 0.82–0.85 [17,18,23,25].

#### 2.3.3. Dietary Acculturation

The Dietary Acculturation Questionnaire for Filipino Americans (DAQFA) was used to determine dietary acculturation. The DAQFA is a validated questionnaire that measures dietary acculturation among Filipino Americans. It was adapted from the dietary acculturation scale developed for Chinese Americans. The tool has 15 food items and dietary behaviors that measures two scales: Filipino dietary acculturation (DAQFAf) and Western dietary acculturation (DAQFAw) [18].

The responses to the DAQFA are listed as yes or no based on the participants’ dietary practices in the past month prior to completing the questionnaire. A higher score in the Filipino scale is indicative of the maintenance of traditional Filipino eating pattern and a higher score in the Western scale reflects adaptation to Western eating pattern. In a previous study, the Cronbach’s alpha coefficient was 0.79 [18]. For this study, the Cronbach’s alpha was found to be 0.74.

#### 2.3.4. Dietary Intake

The Block’s Brief Food Frequency Questionnaire (BFFQ) was used to estimate dietary intake. The Block Food Frequency Questionnaire was originally developed by Dr. Block and obtained through her company NutritionQuest. The BFFQ provides estimated intake of usual and customary foods [26]. The BFFQ is a 70-item questionnaire and takes about 20 min to complete. It is a condensed version of the previously validated full length Block FFQ which was developed from the National Health and Nutrition Examination Survey (NHANES) III dietary recall data. Validation of the condensed version has shown that it is comparable to the full-length version [27]. The tool is designed to be self-administered. However, participants who needed assistance were helped. Individual portion size was asked, and pictures were provided for quantification [26].

NutritionQuest analyzed completed questionnaires and calculated nutrients. The analyzed data was then returned to the researcher. The nutritional variables of interest for this study were the measurements of daily caloric intake, percentage fat intake and percentage carbohydrate intake.

#### 2.3.5. Anthropometric Measurements

The anthropometric measurements that were obtained include BMI and waist circumference. BMI was calculated using height and weight. All measurements were done by one of the authors (PV) to ensure reliability.

The participants’ height and weight were measured without shoes. Height was measured to the nearest 0.25 in using a portable stadiometer. Weight was measured to the nearest 0.1 pound (lb) using digital weighing scale. All participants only had one layer of clothing and pockets were emptied. The BMI was calculated using the National Heart Lung and Blood Institute (NHLBI) BMI calculator [28]. The current guidelines define normal BMI as 18 kg/m^2^ to <25 kg/m^2^, overweight as, BMI ≥ 25 kg/m^2^ to ≤30 kg/m^2^ and obesity as ≥30 kg/m^2^ [29].

Waist circumference was measured to the nearest inches (in) using a non-stretchable measuring tape. Waist measurement was obtained at the level of the umbilicus at the end of normal exhalation. The tape measure was parallel with the floor and snug but not constricting. Increased health risks are associated with waist circumference >35 inches or 88 cm in women and >40 inches or 102 cm in men [30,31].

### 2.4. Statistical Analysis

All data analyses were computed using SPSS 21. Descriptive statistics were used to summarize the acculturation scores, dietary acculturation scores and sociodemographic characteristics of the sample. Pearson’s correlation was used to investigate the relationship between acculturation and dietary acculturation, dietary acculturation and dietary intake and dietary acculturation and anthropometric measurements. A value of *p* < 0.05 was considered statistically significant.

## 3. Results

### 3.1. Sample Characteristics

The socio-demographic characteristics of the participants are presented in Table 1. The total sample (*N* = 210) consisted of 134 (63.8%) female participants and 76 (36.2%) male participants. The majority of the participants were married (74.3%) and had a college degree or higher (82%). Seventy-seven percent were currently employed with 42% working in healthcare related occupations. More than half (55.7%) had an individual income of $50,000 or above with the largest percentage (21.9%) earning $75,000–99,999. The two factors influencing dietary acculturation that were evaluated included the perception of availability and affordability of traditional food. The majority of the participants found Filipino food and ingredients to be available (95.7%) and affordable (89%).

The mean age of the participants was 46.6 years (SD = 13.6, range = 18–74 years). Migration specific data showed that the mean length of residency in the U.S. was 17.6 years (SD = 9.2, range = 1–49 years) and the mean age upon immigration was 28.62 years (SD = 11.8, range = 1–62 years).

**Table 1:** Socio demographic variables.

Sociodemographic Characteristics	*n* = 210 (%)
Gender Male	76 (36.2%)
Female	134 (63.8%)
Marital Status Single	33 (15.7%)
Married	156 (74.3%)
Separated/Divorced	12 (5.7%)
Widow/widower	7 (3.3%)
No answer	2 (1%)
Education Grade school	0
Some high school	7 (3.3%)
High school graduate	9 (4.3%)
Some college	22 (10.5%)
College graduate	145 (69%)
Graduate school	27 (12.9%)
Employed Yes	163 (77.9%)
No	44 (20.7%)
No answer	3 (1.4%)
Occupation Healthcare professional, nursing	69 (32.9%)
Healthcare professional, non-nursing	11 (5.2%)
Healthcare assistive personnel	17 (8.1%)
Non-healthcare	65 (31%)
Not employed	33 (15.7%)
Retired	8 (3.8%)
No answer	7 (3.3%)
Individual Income less than $25,000	20 (9.5%)
$25,000–49,999	41 (19.5%)
$50,000–74,999	33 (15.7%)
$75,000–99,999	46 (21.9%)
$100,000–124,999	23 (11%)
over $125,000	15 (7.1%)
No answer	32 (15.2%)
Perception of availability of Filipino food/ingredients	
Available	95.7%
Not easily available	4.3%
Perceptions of affordability of Filipino food/ingredients	
Affordable	89%
Not affordable	11%

### 3.2. Anthropometric Measurements

The BMI and waist circumference data are presented in Table 2. Of the total sample, 54.3% had a normal BMI and 45.7% were overweight or obese. Among the female participants, 63.4% had a normal BMI and 36.6% were overweight or obese; 76.1% had a normal waist circumference and 23.9% were considered at increased risk (>35 in). Among the male participants, 38.2% had a normal BMI and 61.81% were overweight or obese; 80.3% had a normal waist circumference and 19.7% were considered at increased risk (>40 in).

**Table 2:** BMI and waist circumference.

Anthropometric Measurements		Total (*n* = 210)	Female (*n* = 134)	Male (*n* = 76)
Body Mass Index	Mean (kg/m^2^)	25.4	24.7	26.7
	Normal (%)	54.3%	63.4%	38.2%
	Overweight (%)	36.2%	30.6%	46.1%
	Obese (%)	9.5%	6%	15.8%
Waist Circumference	Mean (in.)		32.3	35.9
	Normal (%)		76.1%	80.3%
	Increased risk (%)		23.9%	19.7%

### 3.3. Acculturation and Dietary Acculturation

The mean acculturation level using the ASASFA was 2.9 (SD = 0.49) on scale of 1–5. The mean Filipino dietary acculturation score was 4.1 (SD = 1) on a scale of 0–5 and the mean Western dietary acculturation score was 6.6 (SD = 2.4) on a scale of 0–10. Participants who were more acculturated had a lower Filipino dietary acculturation. Acculturation was not significantly related with Western dietary acculturation.

### 3.4. Correlations between Acculturation, Dietary Acculturation, Dietary Intake and Anthropometric Measurements

The correlations between ASASFA, DAQFAf, DAQFAw, dietary intake and anthropometric measurements are presented in Table 3. Acculturation level was not significantly correlated with caloric intake (*p* = 0.516), fat intake (*p* = 0.264) and carbohydrate intake (*p* = 0.635). Filipino dietary acculturation was not significantly correlated with caloric intake (0.374), fat intake (*p* = 0.837) and carbohydrate intake (*p* = 0.929). However, participants who had a higher Western dietary acculturation had a higher caloric intake (*p* = 0.005) and fat intake (*p* = 0.025). Western dietary acculturation was also found to have a negative correlation with carbohydrate intake (*p* = 0.206), participants who had a higher Western dietary acculturation had a lower carbohydrate intake.

The participants’ acculturation level was not related to BMI (*p* = 0.730) and waist circumference (*p* = 0.241). Filipino dietary acculturation level was not related to BMI (*p* = 0.997) and waist circumference (*p* = 0.771). However, the participants who had a higher Western dietary acculturation had a higher BMI (*p* = 0.003) and waist circumference (*p* = 0.049).

**Table 3:** Correlations between Acculturation Scale, Dietary Acculturation Scale (Filipino, Dietary Acculturation Scale (Western), Dietary Intake and Anthropometric Measurements.

	ASASFA (*p* Value)	DAQFAf (*p* Value)	DAQFAw (*p* Value)
Calories	0.045 (0.516)	0.062 (0.374)	0.193 * (0.005)
Fat	−0.077 (0.264)	0.014 (0.837)	0.154 * (0.025)
Carbohydrates	−0.033 (0.635)	−0.006 (0.929)	−0.172 * (0.013)
Body Mass Index	−0.023 (0.744)	0.071 (0.304	0.216 * (0.002)
Waist circumference	−0.091 (0.189)	0.064 (0.358)	0.161 * (0.019)

* Denotes findings that reach statistical significance. Significance established at the *p* < 0.05 level; ASAFA—Acculturation Scale for Filipino Americans; DAQFAf—Dietary Acculturation Questionnaire for Filipino Americans Filipino Scale; DAQFAw—Dietary Acculturation Scale for Filipino Americans Western Scale.

## 4. Discussion

The aim of the study is to describe the acculturation and dietary acculturation levels and investigate the relationship between dietary acculturation with dietary intake and health status indicators in a sample of first generation Filipino immigrants. The results indicate that the levels of acculturation and Filipino dietary acculturation do not relate to dietary intake and anthropometric measurements. The most important finding was that Western dietary acculturation was correlated with increased caloric and fat intake and increased BMI and waist measurement.

The relationship between Westernization of diet and increased dietary intake has been documented by other studies on Asian immigrants. Western diet is characterized by bigger servings, increased fast food and increased meat consumption [17,18,32,33,34]. This unhealthy dietary change among immigrants can result in increased risk factors for chronic diseases.

There is a negative correlation of carbohydrate intake with Western dietary acculturation. Rice remains the staple food of Filipino American immigrants and the major source of carbohydrates [16,17,35]. A few studies on dietary changes among the Asian American population have shown decreased rice and carbohydrate intake with Westernization of diet [16,36,37].

In comparison to a previous study on Filipino immigrants in North Carolina, a similar relationship was found between Western dietary acculturation and increased BMI and waist circumference [18]. The participants who had higher Western dietary acculturation had higher intake of fats and sugars and increased BMI and waist circumference. The same study found that participants who had higher Filipino dietary acculturation had lower dietary intake of fat and sugar, which was not found in this current study [18].

Although Filipino dietary acculturation was not related to increased anthropometric measurement or increased caloric, fat or carbohydrate intake, it also did not show a correlation in decreasing health risk factors. One theory in dietary changes with immigration is called festival food syndrome. Festival food syndrome theorizes that immigrants are eating foods that were reserved for celebrations or special occasions in the home country but are now being consumed more frequently in the host country. These festival foods are usually more rich and high-caloric and have become reflective of “traditional” food in the process of acculturation [38]. It is a possibility that those who have a high Filipino dietary acculturation, more festival foods are being consumed, thus eliminating the protective qualities of the traditional diet.

The acculturation level of the participants in this study indicated that there is emerging biculturalism among the immigrants in NJ. The participants’ acculturation level in this study was similar to the acculturation level among first generation Filipino immigrants in California with a similar participant immigration profile [17].

The results showed a high Filipino dietary acculturation level and a moderate Western dietary acculturation level. The high Filipino dietary acculturation among the participants could be attributed to the high availability and affordability of Filipino foods. The availability and affordability of the traditional food is a common environmental determinant in dietary acculturation [2]. There are several Filipino grocery stores and restaurants in Northern NJ where there is a higher concentration of Filipino immigrants. This is reflected by the high percentage of participants who found Filipino food and ingredients available (95.7%) and affordable (88.6%).

Participants who were more acculturated consumed less Filipino food. This result is consistent with the findings of a previous study [18]. Studies have shown that exposure to host culture may lead to changes in diet. This could be a result from changes in beliefs, attitudes and values ascribed to traditional food. Exposure to new food supply could also affect taste preferences and changes in procurement and preparation of food [2].

In this study, the mean BMI and waist circumference were similar to the study findings in Southern California with a mean BMI = 25 kg/m^2^, men = 26.42 kg/m^2^ and women = 24.69 kg/m^2^ and mean waist circumference for men = 32.65 inches, women = 32.7 inches [17]. However, the findings in this study was higher than the study done in North Carolina with a mean BMI = 23.8 kg/m^2^, men = 24.7 kg/m^2^ and women = 23.5 kg/m^2^ and the mean waist circumference for men = 33 inches, women = 29.3 [18].

There is an increased percentage of Filipino immigrants in the U.S. with a BMI > 25, especially among men. Although the majority of the participants had a normal waist circumference, Filipino immigrants have been found to have significantly higher visceral adiposity even at normal BMI and waist circumference [15]. Asians generally have a higher percentage of body fat and visceral adipose tissue than Caucasians of the same age, sex and BMI. Even below the existing BMI cut-off point of 25, the proportion of Asian people with risk factors for type 2 diabetes and cardiovascular disease is substantial. The World Health Organization (WHO) has recommended the use of Asian cut-off points for both BMI and waist circumference to define overweight/obesity in the Asian population which allows for a more accurate identification of health risk in the population [31,39].

## 5. Strengths and Limitations

To the best knowledge of the authors, this is the first study to investigate the correlation between acculturation, dietary pattern and anthropometric measurements among Filipino immigrants in New Jersey. The results of this study will add to the scarce body of knowledge on Filipino immigrant health.

One of the strengths of the study is the use of an ethnic-specific acculturation scale, ASASFA, instead of acculturation proxy measures such as length of stay in the U.S. and age at migration to the U.S. in determining acculturation level. In addition, the actual measurements of height, weight and waist circumference, instead of self- reported measurements, were used in the study.

The study does have some limitations that have to be taken into consideration. The use of a convenience sample and the size of the sample (*n* = 210) limit the generalization of the findings. Furthermore, the participants were limited to certain areas of Northern NJ and, therefore, findings cannot be generalized to all the Filipino immigrants in the USA.

Dietary intake was determined using self-reported food frequency questionnaire. Although frequency and portions were included in the BFFQ, the participants may have difficulty in recalling food intake [35]. The BFFQ is lengthy, taking at least 20 min to answer and even longer for the elderly. This could have affected the participants’ focus. The participants may have answered the questionnaire in a hurried manner. The BFFQ also did not include commonly eaten Filipino dishes. These factors may have decreased the accuracy in measuring the dietary intake.

## 6. Conclusions

This study provides insights on the health status and dietary patterns of first generation Filipino American immigrants in NJ. Based on the literature and the findings of this study, a high percentage of Filipino American immigrants are overweight or obese. This study also provides information on the dietary pattern changes that occur among Filipino American immigrants which include Westernization of diet and increased caloric and fat intake.

It is important for healthcare providers taking care of Filipino American immigrants to do a culturally tailored dietary assessment. It is also vital to educate the population on the increased health risks resulting from Westernization of diet and increased fat intake. Moreover, there is a need to engage newer Filipino American immigrants earlier on regarding dietary counselling to maintain healthy weight and keep healthy traditional practices. In addition, it is also beneficial to provide more acculturated Filipino immigrants with nutritional education focusing on healthier bicultural dietary practices.

The increasing overweight/obesity and prevalence of chronic diseases such as HTN and DM among Filipino immigrants is a cause for public health concern. Future research using the WHO Asian BMI and waist circumference cut-off points would provide a more accurate assessment of the prevalence of overweight/obesity in this population. This study provides evidence of the overweight/obesity status and some of the dietary risks factors that could contribute to the alarming disparity in health among Filipino immigrants. The findings of this study can be used in implementing community health interventions and conducting further research to improve the dietary practices and to decrease overweight/obesity in this population, thereby decreasing risks of chronic illness.
